# Scalable Jet-O-Mized Eggshell Membrane Processing for Bioactives Promoting Gut Health

**DOI:** 10.3390/molecules31071217

**Published:** 2026-04-07

**Authors:** Manar Younes, Tamer A. E. Ahmed, Riadh Hammami, Maxwell T. Hincke

**Affiliations:** 1 Department of Cellular and Molecular Medicine, Faculty of Medicine, University of Ottawa, Ottawa, ON K1H 8M5, Canada; myoun141@uottawa.ca (M.Y.); tahmed@uottawa.ca (T.A.E.A.); 2 School of Nutrition Sciences, Faculty of Health Sciences, University of Ottawa, Ottawa, ON K1H 8M5, Canada; riadh.hammami@uottawa.ca; 3 Department of Innovation in Medical Education, Faculty of Medicine, University of Ottawa, Ottawa, ON K1H 8M5, Canada

**Keywords:** eggshell membrane, alkaline hydrolysis, proteins, antibacterial, antioxidant, anti-inflammatory, gut permeability

## Abstract

This study introduces a novel, simplified, and scalable two-step process for generating bioactive eggshell membrane (ESM) formulations by combining jet-O-mizer ultra-fine milling of ESM (yielding JEM biomaterial) with KOH-mediated hydrolysis, achieving ~50% solubilization of proteins and peptides and enabling the first evaluation of ESM-derived bioactives for gut health applications. The soluble protein fraction (SJ) was separated from the whole hydrolysate (WJ), and subjected to simulated gastrointestinal digestion to assess stability and bioavailability. The antioxidant capacities of the JEM-derived material showed a significant 15-fold increase compared to soluble non-hydrolyzed JEM (NJEM). SJ inhibited *E. coli* bacterial growth by 50% within 24 h, compared to the untreated bacterial culture. The formulations demonstrated superior anti-inflammatory properties with lipopolysaccharide (LPS)-induced RAW macrophages, resulting in a 80% reduction in NO production compared to untreated cells. Proteomics analysis of SJ revealed key anti-inflammatory (YBX1, YWHAE) and antimicrobial (OCX36, OC-17, TENP, and histones) effectors whose coordinated activities could modulate gut microbial composition. The permeability of the intestinal barrier model Caco-2 monolayer was not significantly affected by treatment with any JEM-derived formulation, thereby predicting maintenance of intestinal integrity. This study provides safe, novel ESM derivatives with high bioavailability and multifunctional bioactivities, including antibacterial, antioxidant, and anti-inflammatory effects, positioning them as promising candidates for dietary supplements to promote gut health.

## 1. Introduction

Globally, about 100 million tons of unfertilized chicken eggs (1.65 trillion table eggs) are produced annually [1]. Eggshell (ES) and eggshell membrane (ESM) represent approximately 10–12% and 1.02% (wt. %) of the egg structure, respectively [2]. In Canada, approximately 33% of egg production is directed to the processing sector [3], producing considerable ES and ESM waste, which are potentially valuable raw materials for various applications and secondary processing [4]. Structurally, the ESM is a fibrous semi-permeable membrane wrapped around the egg white and embedded in the ES mammillary cones as the innermost shell component [1]. ESM prevents egg white exudation, protects the egg from external pathogens, provides the initial support for ES mineralization and improves the mechanical properties of the egg [1,5]. Chemically, the fibrils of the ESM consist mainly of proteins (∼90%), with low amounts of lipids (∼3%) and carbohydrates (∼2%) [1] (Supplementary Table S1). Approximately 500 proteins have been identified in the ESM [5], with collagens being the most plentiful, along with other proteins and glycoproteins [6], including cysteine-rich ESM proteins (CREMPs) [1], egg white proteins (i.e., ovalbumin, ovotransferrin and lysozyme) and ES matrix proteins (i.e., ovocalyxin-36, ovocleidin-17) [1,5,7]. The ESM’s distinctive physicochemical, biological, compositional, and mechanical properties, as well as its availability, make it a promising biomaterial for diverse biotechnological applications. Transformed ESM has been studied as a natural supplement for the treatment of joint and connective tissue disorders [8,9,10,11,12,13], as well as applications in wound healing [14], tissue regeneration [15] and food packaging [16], which are based on its anti-inflammatory [14], antimicrobial [17], antioxidant [18], and biosorbent [19] properties.

A number of preclinical studies have suggested that isolated ESM could be repurposed as a beneficial dietary intervention due to its immunomodulatory and anti-inflammatory effects, as well as its capacity to modulate the gut microbiota [20,21,22]. Yang and coworkers showed that supplementation with an 8% (*w*/*w*) ESM diet improved survival and body composition in IL-10 knockout mice, a model of IBD. ESM increased gut microbial diversity, restored the Firmicutes/Bacteroidetes ratio, elevated beneficial bacteria (e.g., *Ruminococcus*), and reduced pro-inflammatory *Enterobacteriaceae*. It also boosted butyrate levels and downregulated inflammatory genes (*Il-1β*, *TNF-α*), highlighting its anti-inflammatory potential. They demonstrated that ESM supplementation improved survival, enhanced gut microbial balance, increased butyrate production, and reduced inflammatory markers, supporting its anti-inflammatory and gut health benefits [22]. In another study, Jia and coworkers showed that ESM supplementation in IL10-knockout mice improved body composition, muscle strength, and reduced inflammation, while enhancing microbiota diversity and short-chain fatty acid (SCFA) production, suggesting its potential for cachexia prevention [20]. ESM may be effective in obesity management, as ESM supplementation in mice fed a high-fat diet lowered plasma triglycerides and liver cholesterol, while increasing expression of lipid-metabolism genes, such as carnitine palmitoyltransferase 1A. Microbiota analysis revealed increased relative abundance of the anti-obesity bacterium Lactobacillus reuteri and decreased abundance of inflammation-related bacteria, such as *Blautia hydrogenotrophica* [21]. Rønning and coworkers reported that a diet with 8% ESM increased gut microbiota diversity and lowered TNF-α expression in mice and *in vitro* in THP-1 macrophages. In their human study, dietary ESM reduced the inflammatory marker C-reactive protein (CRP) [23].

The ESM fibers are highly insoluble due to inter- and intra-molecular disulfide bonds, as well as cross-linking of lysine-derived desmosine and isodesmosine. Previous studies [2,14,24,25] used several solubilization approaches to disrupt the highly cross-linked structure of ESM using mechanical and chemical methods or a combination of these techniques. Physical ESM solubilization strategies included heating, pressurization, extrusion, and ultrasound energy [2]. Mechanical size reduction and sieving of ESM to produce particalized ESM (PEM) does not alter its amino acid composition, which remains consistent with that of unprocessed ESM [14]. Enzymatic digestion can also be utilized to degrade ESM [24]. Lee and coworkers estimated that the solubility of ESM proteins could reach 91.4% through an orthogonal test using acetic acid and pepsin, combined with a segmental extraction technique [25].

Increasing the solubility of processed ESM is an important research direction to improve its bioavailability and potential as a high-value dietary supplement to promote positive gut health. This study introduces a novel, simplified, and scalable approach for solubilizing jet-O-mized eggshell membrane (JEM) using mild alkaline hydrolysis to produce bioactive peptides from ESM. Unlike traditional enzyme- or acid-based protocols, this process combines jet-milled ESM with low-concentration KOH treatment under moderate conditions to achieve high solubilization efficiency while preserving bioactivity. The optimized method offers an economical strategy for producing safe, functional ESM formulations with antioxidant, anti-inflammatory, and gut barrier-supporting properties demonstrated in this study, supporting their potential development as high-value dietary supplements for gut health.

## 2. Materials and Methods

### 2.1. Materials

The bacterial strain used in this study was *E. coli* (0157:H7 derived from ATCC^®^ 43888™). Bacterial cultures were maintained and grown using Luria–Bertani (LB) broth (BioShop, Burlington, ON, Canada). The cell lines used in the study included RAW 264.7 macrophages (ATCC^®^ TIB-71™, Lot#70012232) and Caco-2 cells (ATCC, HTB-37). Unless stated otherwise, all additional chemicals and supplies were procured from Fisher Scientific, Ottawa, ON, Canada or MilliporeSigma, Oakville, ON, Canada.

### 2.2. Jet-O-Mized (JEM) Biomaterial

In this study, the material under investigation consisted of ESM powder provided by our industrial partner Burnbrae Farms (BBFs, Lyn, ON, Canada), which was subjected to further processing via a high-efficiency vertical jet mill (Fluid Energy Processing and Equipment Company, Telford, PA, USA) designed for grinding dried powders to 1–50-micron average particle size, to produce JEM. The particle size distribution of the ESM coarse powder supplied by Burnbrae Farms (BBFs) was determined by sieve analysis using a Keck sieve shaker, and the mean particle diameter was calculated using a standard weighted class-midpoint method [26]. Microbiological assessments and chemical analyses of JEM were conducted by Eurofins Environex (Longueuil, QC, Canada), using validated analytical protocols, to quantify microbial contamination and characterize chemical constituents, which provided essential data for quality evaluation. Particle size distribution for a suspension of JEM in 2-propanol was determined using a Beckman Coulter LS Particle Size Analyzer (Beckman Coulter, Inc., Brea, CA, USA).

### 2.3. Alkaline Hydrolysis of JEM

**Figure 1:**
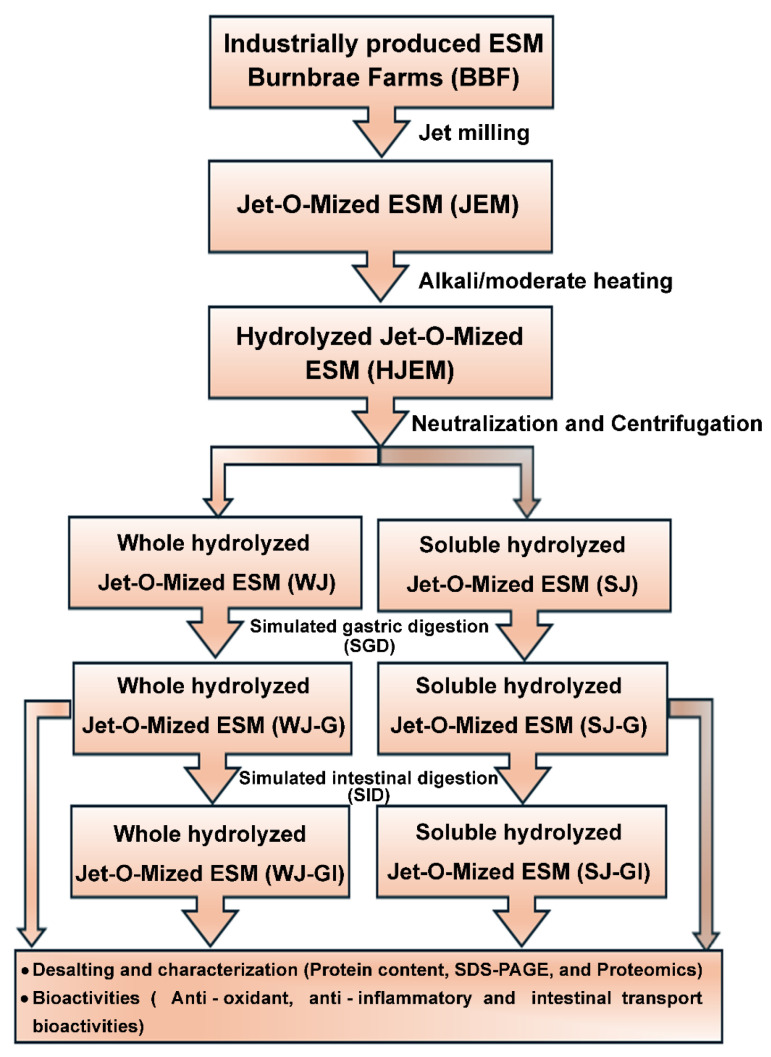
Schematic representation of the JEM processing, purification, characterization, and subsequent *in vitro* assessments.

In preliminary trials, the alkaline hydrolysis of PEM using potassium hydroxide (KOH) under various conditions was investigated to optimize the production of PEM hydrolysate (HPEM, containing bioactive proteins and peptides), including temperature, time, PEM and KOH concentrations, and stirring intensity [27]. Building on that protocol, the current study focused on hydrolyzing JEM with further investigation of the hydrolysis time to achieve a suitable degree of hydrolysis. The aim was to hydrolyze JEM partially to produce HJEM. Thereafter, samples were collected at fixed intervals and analyzed by SDS-PAGE. The reaction was terminated by neutralization with 11.6 M hydrochloric acid, after which the entire hydrolysate (WJ) was centrifuged to separate the soluble fraction (SJ, containing soluble proteins and peptides) from the insoluble pellet. Post-hydrolysis, WJ and SJ were stored at −20 °C for subsequent analysis and processing. The overall processing scheme is summarized in Figure 1. The optimal conditions for producing hydrolyzed-JEM (HJEM) were determined (subject to invention disclosure).

### 2.4. Simulated Gastrointestinal Digestion of JEM Hydrolysate

WJ and SJ were separately subjected to simulated gastrointestinal digestion (SGID), following a standardized method detailed by Minekus and coworkers [28]. This method simulates the physiological conditions of the human gastrointestinal tract and comprises the following two phases: simulated gastric digestion (SGD) and simulated intestinal digestion (SID). In the gastric phase, the JEM formulations were mixed with simulated gastric fluid (SGF) and pepsin, then adjusted to pH 3.0 with concentrated HCl and incubated at 37 °C for 2 h to simulate stomach digestion. At the end of the gastric digestion, samples were collected and stored at −20 °C for subsequent analysis and processing. The resultant simulated gastric metabolites were abbreviated WJ-G and SJ-G. In the intestinal phase, the gastric metabolites WJ-G and SJ-G were mixed with simulated intestinal fluid (SIF), bile salts, and pancreatin, and the pH was adjusted to 7.0 using 10 N NaOH. The mixture was incubated at 37 °C to simulate digestion in the small intestine. The resultant simulated intestinal metabolites WJ-GI and SJ-GI were collected after 4 h of simulated digestion and stored at −20 °C for subsequent analysis and processing.

### 2.5. Desalting/Concentration of JEM Formulations

Before proceeding with the evaluation of the bioactivities of the various soluble JEM formulations (SJ, SJ-G, SJ-GI, WJ-G and WJ-GI), desalting was conducted to remove residual KCl formed upon neutralization of the HJEM with HCl. Centrifugal filter units (Millipore™ Amicon™ Ultra-15 Centrifugal Filter Units, MilliporeSigma, Oakville, ON, Canada, MWCO: 3 KDa) were used to remove the salt and concentrate the JEM formulations. The manufacturer’s procedures were followed, which involved loading 15 mL of the clear supernatant into the desalting column and centrifuging at 5500 RPM for 40 min at 4 °C (16 Lynx 4000 Centrifuge). To enable sample desalting and washing, the sample volume in the column was brought to 15 mL with either phosphate-buffered saline (PBS) or Dulbecco’s Modified Eagle medium (DMEM; Gibco, Grand Island, NY, USA), and the column was then re-centrifuged. This washing step was repeated three times to ensure the thorough removal of KCl, and replacement with suitable physiological media. At the end of the desalting process, protein solutions retained in the upper compartment of the column were collected, analyzed for their protein content, and stored at −20 °C for subsequent processing.

### 2.6. Protein Quantification

The outcomes of the hydrolysis and digestion reactions were assessed using the Bicinchoninic acid (BCA) assay and SDS-PAGE before and after desalting/concentration.

**BCA Assay:** The soluble protein content of the hydrolysate SJ and the metabolites SJ-G, SJ-GI, WJ-G, and WJ-GI were quantified using the Pierce BCA Protein Assay Kit reagent (Thermo Fisher Scientific, Rockford, IL, USA), as per the manufacturer’s instructions. The absorbance of the developed color was measured at 562 nm and corrected for absorbance at 600 nm using a microplate reader (BioTek Eon, BioTek, Winooski, VT, USA) operating in the dual-wavelength mode [14]. Bovine serum albumin (BSA) standard curves were prepared in appropriate solvents (neutralized KOH, SGF or SIF), in order to calculate the protein concentration of samples.

**SDS-PAGE** was used to visualize the apparent molecular weight distribution of soluble protein constituents of the JEM formulations, using precast polyacrylamide gels (Blot^TM^ 4–12% Bis-Tris Plus, Thermo Fisher Scientific, USA) and prestained protein molecular weight markers (Blue Standards 161-0373). The gel was run (Blot^TM^ MES SDS Running Buffer, Invitrogen, Carlsbad, CA, USA) for 20 min at 220 volts, followed by staining with 0.25% Coomassie Brilliant Blue R-250, and destaining (50% *v*/*v* Methanol, 10% *v*/*v* acetic acid) to visualize the protein bands [5].

### 2.7. Protein Characterization

#### 2.7.1. LC/MS/MS-Based Proteomics Analysis

Hydrolyzed ESM material (SJ and WJ-G) was processed for proteomics at the Eastern Quebec Genomics Center for LC–MS/MS profiling using RP-nanoLC coupled to an Agilent 1200 nanopump and an AB Sciex 5600 mass spectrometer equipped with a nanoelectrospray ionization source (nano-ESI). Data acquisition (ES–MS/MS) was performed in Analyst 1.6. Peak lists were generated in ProteinPilot 4.5 and searched against REF_GGallus_cUP000000539_20220913 and contaminants_thegpm_20200924 (27,599 entries) using Mascot 2.4.0 and X!Tandem (CYCLONE). Searches assumed fixed Cys carbamidomethylation and variable deamidation (N/Q), N-terminal Gln→pyro-Glu, and Met/Pro oxidation [5,14].

#### 2.7.2. Protein Identification

Datasets were curated in Scaffold 5.3.3 against the NCBI Gallus gallus proteome. Identity confirmation used BLASTP (v2.17.0) against the non-redundant database, based on coverage, E-value, and percent identity, with redundant entries and contaminant proteins excluded. Peptides were accepted at ≥95% probability; proteins were filtered at 1% FDR with at least 1 unique peptide identification [5].

#### 2.7.3. Bioinformatics Analysis

Gene Ontology (GO) term enrichment profiling of proteins detected in SJ and WJ was conducted using the Database for Annotation, Visualization and Integrated Discovery framework (Knowledgebase v2025_1). Significantly enriched GO terms (*p* < 0.05) were prioritized based on *p*-value-driven clustering. Each GO term was associated with an EASE score (a modified Fisher Exact *p*-value reflecting enrichment) that reflects enrichment stringency. Enrichment mapping encompassed both Biological Process (GOTERM_BP) and Molecular Function (GOTERM_MF) ontological domains [5]. Proteins assigned to each enriched GO term were subsequently analyzed using the STRING platform (http://string-db.org/) to delineate all putative interaction networks, incorporating evidence-based interaction scores based on experimental data, database annotations, co-expression metrics, and computational predictions to resolve both direct and indirect functional associations within each GO-defined protein set.

### 2.8. Assessment of Bioactivities of the Developed JEM Formulations

Based on the concentration of soluble proteins and peptides obtained from each formulation after desalting, serial dilutions were prepared, and their activities were measured.

#### 2.8.1. Antibacterial Activity

Antibacterial activity of serially diluted SJ against *E. coli* was measured using the broth dilution assay. SJ was selected for antimicrobial activity because sufficient material was available to evaluate dose-dependent antimicrobial effects at higher concentrations using *E. coli* O157:H7 as a representative intestinal pathogen. Dilutions of SJ to 1.25, 2.5, 5 and 10 mg/mL were prepared in Luria–Bertani (LB, BioShop, Canada) broth, mixed 1:1 with bacteria (2 × 10^5^ CFU/mL) in a 96-well microplate, and then incubated at 37 °C in a microplate reader, with OD600 measured at time zero, and hourly for 24 h. Each treatment was replicated in 5 wells to verify reproducibility. Wells containing bacteria without SJ served as a negative control, while wells containing 0.62 µg/mL gentamicin (Sigma-Aldrich) served as a positive control. Blank wells contained only LB broth. Growth curves were obtained by plotting OD600 against time for each treatment. The antibacterial effect was determined by comparing the growth curves of the treated samples with those of the controls [14].

#### 2.8.2. Total Antioxidant Activity

The Trolox equivalent antioxidant capacity (TEAC) of non-hydrolyzed JEM (NJEM) and the JEM formulations SJ, SJ-G, SJ-GI, WJ-G and WJ-GI at the concentrations 1.25, 2.5, 5 and 10 mg/mL was measured using the Total Antioxidant Power Kit (Oxford Biomedical Research, Rochester Hills, MI, USA, Product Number: TA02), as per the manufacturer’s instructions. NJEM, JEM formulations and Trolox standards were diluted 1:40 in the provided dilution buffer. An aliquot of 100 µL JEM formulations or Trolox standards was added to a 96-well plate prior to a reference dual-wavelength absorbance measurement at 450 and 600 nm. Copper solution (25 µL) was added to each well, and the mixture was incubated for 10 min at room temperature. Next, 25 µL stop solution was added to each well, and the absorbance at 450 and 600 nm was measured a second time. The net absorbance was calculated by subtracting the reference absorbance readings [27]. The dilution buffer served as a reagent blank.

#### 2.8.3. *In Vitro* Anti-Inflammatory Activity

The anti-inflammatory activity of JEM formulations was assessed by evaluating their impact on the accumulation of the pro-inflammatory mediator nitric oxide (NO) in lipopolysaccharide (LPS)-stimulated RAW 264.7 macrophages.

##### RAW 264.7 Macrophage Cell Line and Growth Conditions

RAW 264.7 macrophages were cultured in DMEM complete culture medium (DMEM-CCM): DMEM enriched with 10% heat-inactivated fetal bovine serum (FBS; Corning, collected in Canada, processed in USA), 100 U/mL penicillin, and 100 mg/mL streptomycin (Pen-Strep; Gibco, NY, USA), and 2 mM glutamine (Glutamax, Gibco, NY, USA) in a humidified atmosphere containing 5% carbon dioxide (CO_2_), at 37 °C. The cells were incubated in a 25 cm^2^ flask (Corning Inc., Corning, NY, USA) and subcultured to a 75 cm2 flask (Corning) at approximately 70% confluency, with an initial seeding density of 2 × 10^4^ cells/mL. Cells were maintained by changing the culture medium three times weekly and subcultured at ~70% confluency using 0.25% trypsin-EDTA (Ethylenediaminetetraacetic acid) (Gibco, NY, USA). Pro-inflammatory effects were induced in the macrophage cultures using LPS (*Escherichia coli* O55:B5; MilliporeSigma).

##### Cell Viability Assay

To determine the optimal LPS concentration for inducing pro-inflammatory effects in RAW 264.7 macrophages without compromising cell viability, a range of LPS concentrations was evaluated, using the alamarBlue^®^ assay to assess cell health. Briefly, RAW 264.7 cells were seeded at a seeding density of 25 × 10^3^ cells/well into 48-well culture plates (Falcon, *n* = 3 per treatment) and incubated for 24 h to facilitate cell adhesion and proliferation. The conditioning media was then replaced with fresh media containing various concentrations of LPS (1, 5, 10, and 20 µg/mL) and incubated for an additional 24 h. The media was replaced with fresh DMEM-CMM containing 10% alamarBlue^®^ reagent (Thermo Fisher Scientific, USA), and the cells were incubated for 1 h at 37 °C. After incubation, 150 µL from each well was transferred to a microcentrifuge tube and centrifuged at 4 °C (13,000 rpm, 5 min). The supernatant was then transferred to a 96-well microplate (Corning™ Clear Polystyrene 96-Well Microplates), and fluorescence intensity was measured using Tecan Microplate Reader Spark^®^ (Grödig, Salzburg, Austria) at excitation and emission wavelengths of 560 nm and 590 nm, respectively. The negative control was culture media containing LPS without cells, while the positive control was cells cultured in the absence of LPS. Normalized cell viability for each LPS concentration was determined using the following equation:

(1) $$Normalized\;cell\;viability\;=\;\frac{\left({Fluorescence}_{LPS\;treatment}-{Fluorescence}_{Negative\;control}\right)}{{(Fluorescence}_{Positive\;control}-{Fluorescence}_{Negative\;control})}\times 100\%$$

The same protocol was applied to evaluate the effect of JEM formulations at concentrations of 1.25, 2.5, 5, and 10 µg/mL on the viability of RAW 264.7 macrophages induced with 1 µg/mL LPS after 24 h of treatment. The negative control was culture media containing LPS and JEM formulations without cells, and the positive control was cells cultured with LPS and without exposure to JEM formulations. Normalized cell viability for each JEM treatment was determined using the following equation:

(2) $$Normalized\;cell\;viability\;=\;\frac{\left({Fluorescence}_{JEM\;treatment}-{Fluorescence}_{Negative\;control}\right)}{{(Fluorescence}_{Positive\;control}-{Fluorescence}_{Negative\;control})}\times 100\%$$

##### Nitric Oxide Production Assay

RAW 264.7 cells were seeded at a seeding density of 25 × 10^3^ cells/well into 48-well culture plates (Falcon, *n* = 3 per treatment) and incubated for 24 h to facilitate cell adhesion and proliferation. The conditioning media was then replaced with fresh media containing 1 µg/mL LPS along with JEM formulations SJ, SJ-G, SJ-GI, WJ-G and WJ-GI at concentrations of 1.25, 2.5, 5 and 10 mg/mL, and incubated for an additional 24 h. An aliquot (150 µL/well) of culture media was collected, centrifuged (4 °C, 13,000 rpm) for 5 min and added to a 96-well plate. The accumulation of NO in the culture supernatant was detected using the Griess Reagent System (Promega, Madison, WI, USA) following the manufacturer’s instructions. Cells treated with JEM formulations and without LPS were used as a negative control, while cells with LPS without JEM formulations served as a positive control. Accumulated NO levels were determined using a standard curve of known concentrations of nitrite. NO (%) by cells compared to the positive control was determined from the standard curve using the following equation:

(3) $$Production\;of\;NO\;\left(\%\right)=\frac{\;({NO}_{JEM\;treatment}-{NO}_{negative\;control)}}{({NO}_{Positive\;control}-{NO}_{Negative\;control)}}\times 100\%$$

#### 2.8.4. Effect of JEM Formulations on Intestinal Barrier Function

The effect of the JEM formulation on modulation of intestinal barrier function was assessed by measuring changes in transepithelial electrical resistance (TEER) and paracellular permeability in the differentiated Caco-2 monolayer.

##### Caco-2 Cell Line and Growth Conditions

Caco-2 cells were cultured in DMEM-CCM at 37 °C in a humidified atmosphere containing 5% CO_2_. The conditioning media was changed every other day, and cells were sub-cultured at approximately 70% confluency using trypsin-EDTA.

##### Preparation of Cell Monolayers

Caco-2 cells were seeded at a density of 25 × 10^3^ cells/insert (*n* = 3 per treatment) onto Thincert cell culture insert (0.4 μm pore size, Greiner bio-one, Frickenhausen, Germany) and placed in 12-well plates. Each insert was maintained in DMEM-CCM, 0.5 mL in the apical compartment and 1 mL in the basolateral compartment. The medium was changed every other day, and the formation of a confluent monolayer was monitored by measuring the TEER of the Caco-2 monolayer (Epithelial Vol-Ohm meter, Milli cell^®^ ERS-2, MilliporeSigma, Oakville, ON, Canada). When the TEER values reached a stable reading of approximately 300 Ω·cm^2^ (about 3 weeks), indicating the formation of tight junctions, the cells were considered ready for treatment with the test material.

##### Assessment of the Effect of JEM Formulations on TEER of Caco-2 and Transport of FITC Through the Monolayer

The JEM formulations were tested at the following four different concentrations: 1.25, 2.5, 5 and 10 mg/mL. The material was diluted with sterile PBS containing 100 U/mL penicillin and 100 mg/mL streptomycin to achieve the desired concentrations. Once the TEER reached 300 Ω·cm^2^, the conditioning medium in the apical compartment was carefully removed and replaced with 250 μL of the tested JEM formulation diluted in PBS, and the volume was completed to 0.5 mL with 250 μL of DMEM-CCM containing 1 mg/mL Fluorescein isothiocyanate–Carboxymethyl–Dextran (FITC-CM-Dextran, FD-4) (MilliporeSigma, Oakville, ON, Canada). The basolateral medium was replaced with 1 mL of fresh DMEM-CCM [29]. Each concentration of the JEM formulations was tested in triplicate to ensure reproducibility. TEER measurements were taken at zero time (pre-treatment) and 24 h (post-treatment) to evaluate the effect of JEM formulations on the integrity of the Caco-2 cell monolayer. The measurements were performed in triplicate, and the values were recorded. TEER values were normalized to the initial reading (pre-treatment TEER value) to account for any variations in baseline resistance between wells. The negative control consisted of Caco-2 cells cultured in DMEM-CCM alone, without any test material, to account for changes in TEER due to the medium replacement. The positive control was treatment with the known tight junction disruptor ionomycin (IC, 1 µM). To assess the effect of the treatments on the paracellular permeability of Caco-2 cells, a FITC-CM-Dextran permeability assay was performed, in which the polysaccharide dextran serves as a carrier for the FITC fluorescent dye, allowing tracking of its movement across the Caco-2 monolayer [29]. At zero time, 100 µL of basolateral medium was collected from each well and transferred to a black 96-well microplate. Fluorescence intensity was measured using a Tecan Spark^®^ Microplate Reader (Grödig, Austria) at 490 nm excitation and 520 nm emission. This was repeated after 24 h of incubation with the JEM formulations. The fluorescence intensity of FITC that crossed the Caco-2 monolayer in treatment and control wells was measured and evaluated.

### 2.9. Statistical Analysis

Statistical analyses were performed using GraphPad Prism (version 10). Differences among the treatment groups and the control group were analyzed using One-Way ANOVA. Data are presented as the mean ± standard error derived from three independent experiments conducted in triplicate for each assay. For all analyses, a *p*-value < 0.05 was considered statistically significant. Graphs were generated in GraphPad Prism and Adobe Photoshop CS (version 8.0) to ensure a clear representation of data trends and statistical significance.

## 3. Results

### 3.1. Production and Quality Assessment of JEM

JEM represents size-reduced particles of ESM obtained by jet-milling. The ESM coarse powder provided by our industrial partner Burnbrae Farms (BBFs) was analyzed for its particle size distribution using a Keck sieve shaker kit, which revealed: <53 μm (4.8%), 53–106 μm (30.7%), 106–381 μm (63.5%), 381–508 μm (1.1%) and >508 μm (0%) [calculated average particle size of 185 μm]. This ESM powder was processed in a high-efficiency vertical jet mill to produce jet-O-mized eggshell membrane powder (JEM), which was thoroughly evaluated for chemical composition, microbiological profile (Supplementary Table S2) and particle size distribution (Supplementary Figure S1). Chemical composition analysis showed that JEM powder is predominantly composed of proteins, accounting for 82.9% by weight, along with other constituents, including glucosamine, chondroitin, and hyaluronic acid (HA) (Supplementary Table S3). A comprehensive microbiological assessment was conducted to assess the safety and quality of the JEM powder, indicating that JEM is free from harmful bacterial contamination such as *E. coli* and Salmonella, as follows: aerobic colony count (<5 colony-forming units per gram (CFU/g)), total coliforms (<10 CFU/g), *E. coli* (<10 CFU/g) and *Salmonella* spp. (not detected, as verified by the MFLP-29 method). Particle size analysis of JEM revealed that 99% < 20.49 μm, 100% < 47.94 μm, with an average particle size of 5.7 μm.

### 3.2. Hydrolysis of PEM and JEM

Particalized eggshell membrane (PEM, reduced-size particles < 100 µm) [14] was evaluated in preliminary experiments, to investigate various acid- and base-mediated hydrolysis conditions (concentration: 0.125 to 5 N; temperature: 22–55 °C; different stirring intensities and durations: 30 min to 7 days) (unpublished data subjected to invention disclosure agreement). Following a comprehensive evaluation of these parameters, we identified optimized conditions for producing HJEM [27]. In the current study, an optimized protocol was used for alkaline hydrolysis of JEM with KOH to promote the release of bioactive proteins and peptides (exact conditions subject to invention disclosure). SDS-PAGE analysis of the separated supernatant at the start of the hydrolysis process and the clear supernatant separated from the produced hydrolysate after 24 and 48 h of hydrolysis revealed significant changes in protein profile (Supplementary Figure S2). Prior to hydrolysis, the soluble JEM protein pattern showed a faint smear; however, after 24 h of treatment, the HJEM displayed an intense smear, especially at higher molecular weights, indicating increased solubility of degraded proteins and peptides in SDS. After 48 h of hydrolysis, the smear profile shifted to a lower molecular weight pattern (Supplementary Figure S2).

### 3.3. Quantification of Released Soluble Proteins and Peptides

The concentration of soluble proteins in both SJ and WJ was monitored during successive stages of simulated GI digestion (SGID: SGD and SID) using the BCA protein quantification assay and visualization by SDS-PAGE analysis (Supplementary Figure S2). The initial concentration of soluble proteins in the clear desalted portion of both SJ and WJ was 22.9 ± 4.4 mg/mL. Throughout the SGID, the samples were diluted 1:1 twice, first with the components of the SGD reaction and then with those of the SID reaction. As shown in Table 1, SGD of the WJ resulted in a significant increase in soluble protein concentration, from 11.5 mg/mL at the start of the digestion process to 21.6 ± 0.4 mg/mL after 2 h of SGD. In contrast, the SID of WJ showed a less pronounced increase in liberated protein concentration, where it increased from 10.5 mg/mL to 14 ± 1.3 mg/mL. SGID of SJ did not change the concentration of soluble proteins (Table 1).

### 3.4. Protein Characterization

**Figure 2:**
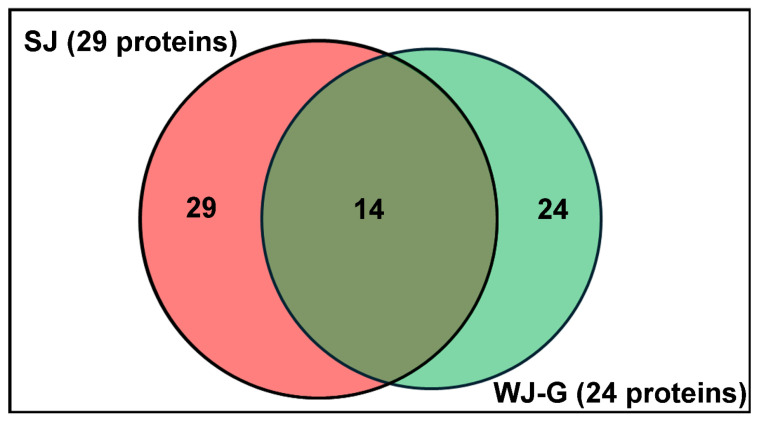
Venn diagram showing the protein constituents of SJ compared to WJ-G.

Thirty-nine (39) proteins in total were identified in JEM-derived soluble formulations, including SJ (29) and WJ-G (24), with 14 proteins common to both (Figure 2 and Table 2).

**Figure 3:**
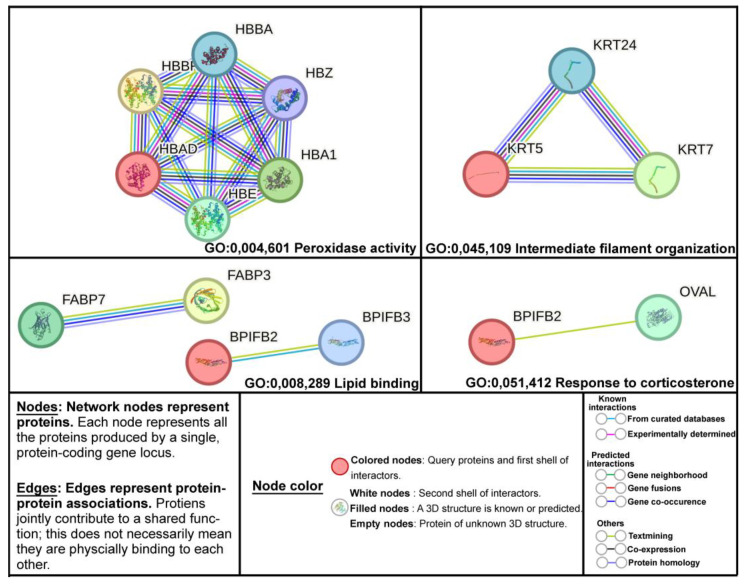
STRING analysis of the identified proteins in SJ and WJ-G, highlighting 4 GO term-based functional clusters. Nodes are grouped by enriched GO categories, illustrating predicted intra- and inter-cluster interactions that may underline coordinated biological activities.

Functional annotation of various JEM formulations using Gene Ontology (GO) term enrichment analysis. The GO term analysis generated the following nine unique functional groups that were significantly enriched (*p* < 0.05): peroxidase activity, lipid binding, protein heterodimerization activity, intermediate filament organization, structural molecule activity, shell calcification, negative regulation of calcium ion export across plasma membrane, response to corticosterone, and positive regulation of cytoplasmic translation (Table 3). The functionalities predicted by the DAVID bioinformatics interface were used as input to STRING to identify all possible protein–protein interactions across different GO terms, including peroxidase activity, lipid binding, intermediate filament organization, and response to corticosterone (Figure 3).

### 3.5. Assessment of Bioactivities of the Developed JEM Formulations

The bioactivities of the developed JEM formulations are summarized in Table 4.

#### 3.5.1. Antibacterial Activity

**Figure 4:**
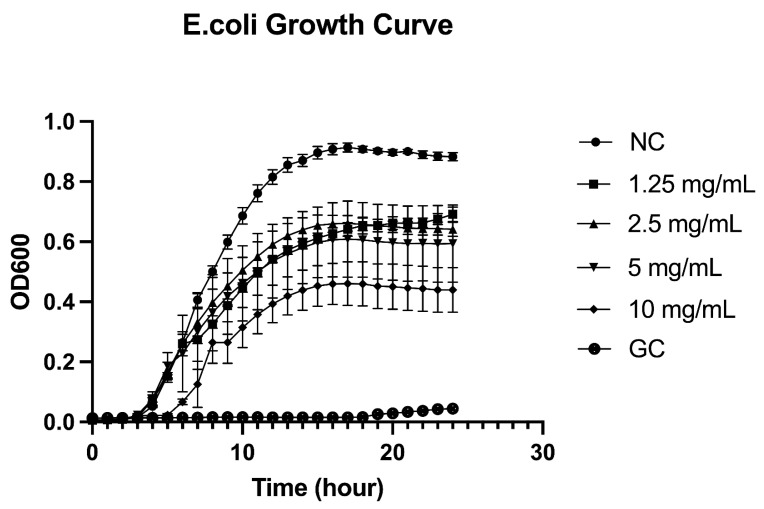
Antibacterial effect of various concentrations of SJ on *E. coli* growth over 24 h. The negative control (NC) is untreated *E. coli*, and the positive control (GC) is *E. coli* culture treated with gentamicin (0.62 µg/mL). The y-axis shows turbidity due to *E. coli* growth measured as OD600. Error bars represent the standard deviation from the mean of five measurements.

The growth inhibition of *E. coli* by SJ was evaluated by measuring OD600 over 24 h at five concentrations (1.25, 2.5, 5, and 10 mg/mL) and compared with a negative control (no treatment) and a positive control (*E. coli* culture treated with gentamicin). At SJ concentrations of 1.25 mg/mL, 2.5 mg/mL and 5 mg/mL, *E. coli* growth was significantly inhibited compared to the control. After 24 h of incubation, the bacteria reached maximum OD600 values of 0.69 ± 0.01, 0.64 ± 0.02, and 0.59 ± 0.13, respectively, which were significantly lower than the control (maximum OD600 of 0.88 ± 0.01). At the highest SJ concentration (10 mg/mL), inhibition of bacterial growth was more pronounced (0.44 ± 0.07 at 24 h), corresponding to 50% inhibition compared to the untreated bacterial culture, with an initial bactericidal effect of 64-times reduction in cell number [5,14] (Figure 4).

#### 3.5.2. Antioxidant Activity

**Figure 5:**
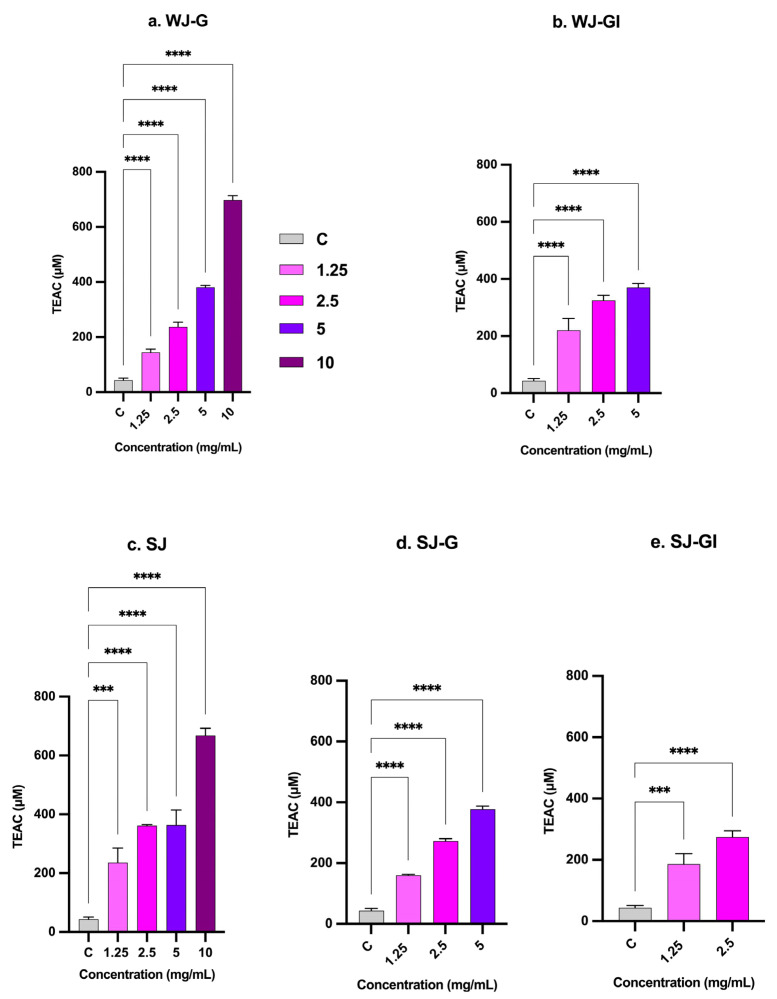
The bar chart illustrates the dose-dependent increase in antioxidant activity of JEM formulations, expressed as TEAC, compared to the control NJEM. Significant differences between treatments and control are marked (***, *p* < 0.001; ****, *p* < 0.0001). Error bars represent the standard deviation from the mean of three independent experiments, with each experiment performed in triplicate.

In this study, the Trolox equivalent antioxidant capacity (TEAC) of JEM formulations was evaluated using the Total Antioxidant Power Kit; total antioxidant activity is expressed as μmol of Trolox equivalents (Figure 5). TEAC was evaluated at various concentrations (1.25, 2.5, 5, and 10 mg/mL) for five JEM formulations (SJ, SJ-G, SJ-GI, WJ-G, and WJ-GI) and compared to non-hydrolyzed JEM (NJEM). At all concentrations, the newly developed JEM formulations showed a significant increase in TEAC compared to the NJEM. At the lowest concentration of 1.25 mg/mL, all formulations showed a minimal but still significant increase in TEAC compared to NJEM, with SJ exhibiting the highest TEAC of 235 ± 50 μM, which is 5-fold that of NJEM. However, as concentration increased, clear patterns of activity emerged: TEAC values increased across all treatments, demonstrating a dose-dependent response. At the highest concentration of 10 mg/mL, the antioxidant capacity of SJ and WJ-G significantly increased, reaching 667 ± 25 μM and 697.7 ± 15.8 μM, which is approximately 15-fold higher than the TEAC of the NJEM (43 ± 7.1 μM).

#### 3.5.3. Anti-Inflammatory Activity

**Figure 6:**
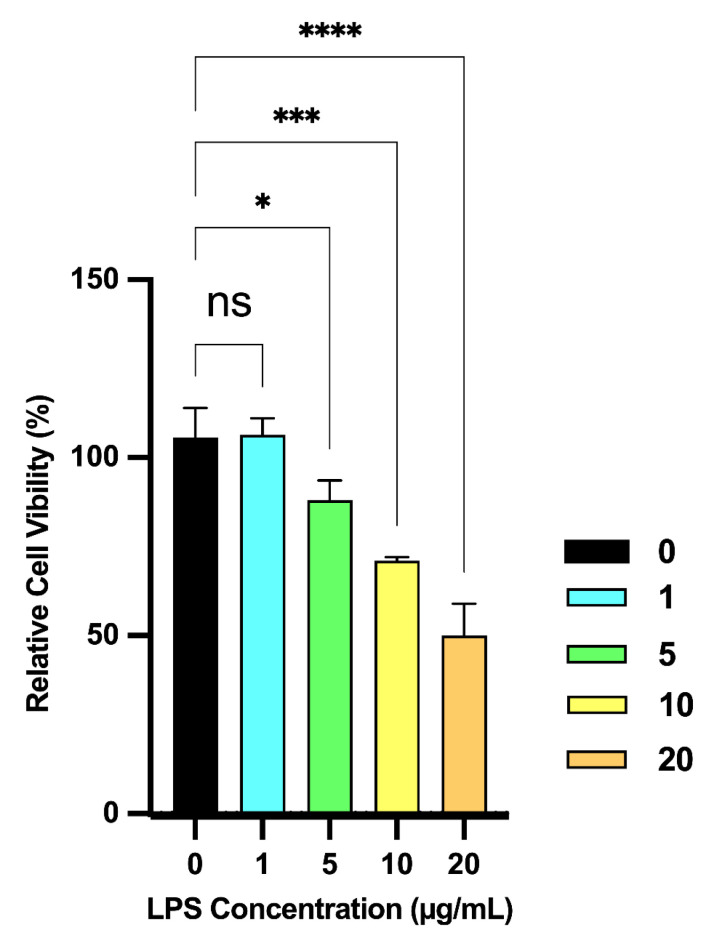
Effect of various concentrations of LPS on the viability of RAW 264.7 macrophages. The impact of various LPS concentrations (1, 5, 10 and 20 µg/mL) was evaluated after 24 h of treatment, compared to the negative control (NC: concentration zero). Significant differences between treatments and control are marked (*, *p* < 0.05; ***, *p* < 0.001; ****, *p* < 0.0001). ns: not significant (*p* > 0.05). Error bars represent the standard deviation from the mean of three independent experiments.

Cytotoxicity of LPS: The effect of various concentrations of LPS (0.1, 5, 10 and 20 μg/mL) on the viability of RAW 264.7 macrophages was assessed (Figure 6). Only the lowest LPS concentration (1 μg/mL) did not show a significant deleterious effect on cell viability after 24 h of treatment.

**Figure 7:**
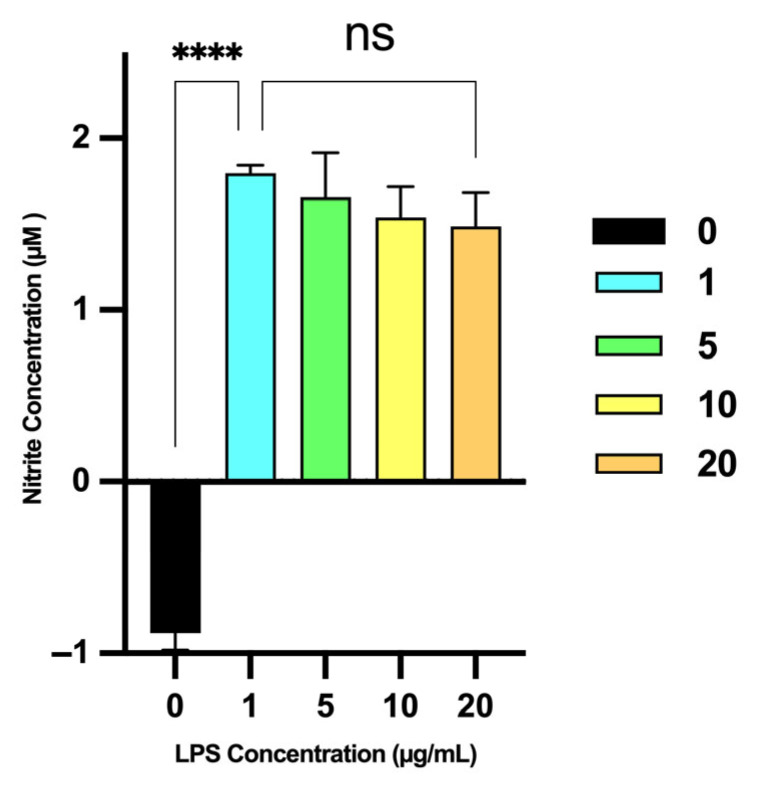
Effect of varying concentrations of LPS on NO production in RAW 264.7 macrophages after 24 h of treatment. Nitrite levels were measured as an indicator of NO production and expressed in µM. Absorbance was measured at 540 nm; minor negative values reflect blank subtraction. ****, *p* < 0.0001, ns: not significant (*p* > 0.05). Error bars represent the standard deviation from the mean of three independent experiments, with each experiment performed in triplicate.

LPS induction of nitric oxide production: The effect of the various LPS concentrations (1, 5, 10 and 20 μg/mL) on NO production in RAW 264.7 macrophages is illustrated in Figure 7. No significant change in the nitrite concentration was recorded upon an increase in LPS concentration from 1 μg/mL (NO: 1.8 ± 0.05 μM) to 20 μg/mL (NO: 1.5 ± 0.2 μM). Based on these results, a concentration of 1 μg/mL LPS was selected for subsequent experiments to induce NO production in RAW 264.7 macrophages, as this concentration yielded the highest NO levels without compromising cell viability.

Impact of JEM formulations on RAW 264.7 macrophages: The effect of various concentrations of SJ, SJ-G, SJ-GI, WJ-G and WJ-GI (1.25, 2.5, 5 and 10 mg/mL) on the viability and NO production in LPS-induced RAW 264.7 macrophages was assessed and compared to a negative control of untreated macrophages. No significant cytotoxicity was observed at different concentrations of various JEM formulations compared to the negative control, as cell viability remained above 80% at all concentrations.

**Figure 8:**
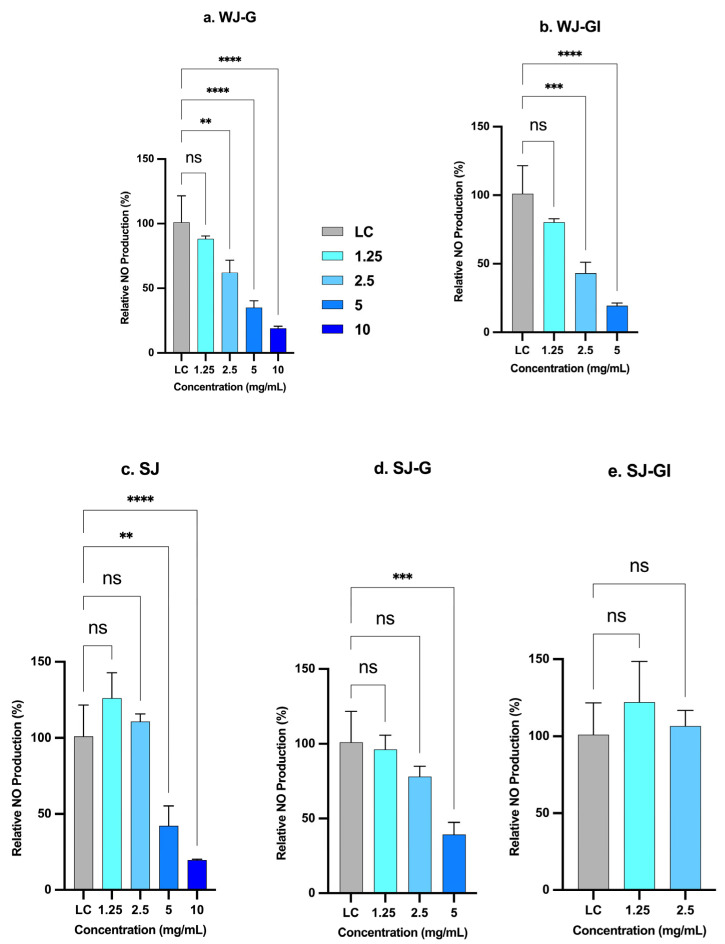
Effect of JEM formulations on the relative production of NO in LPS-stimulated RAW 264.7 macrophages after 24 h of treatment with different concentrations in comparison to the positive control (LC) (0 mg/mL, untreated LPS-induced RAW 264.7 macrophages). Significant differences between treatments and control are marked (**, *p* < 0.01; ***, *p* < 0.001; ****, *p* < 0.0001). ns: not significant (*p* > 0.05). Error bars represent the standard deviation from the mean of three independent experiments, with each experiment performed in triplicate.

Inhibition of nitric oxide production by JEM formulations: The effect of the various concentrations of SJ, SJ-G, SJ-GI, WJ-G and WJ-GI (1.25, 2.5, 5 and 10 mg/mL) on the relative production of NO in LPS-induced RAW 264.7 macrophages was assessed and compared to the untreated positive control of LPS-induced RAW macrophages. Results showed that inhibition of LPS-stimulated NO production was dose-dependent, with higher concentrations yielding a greater reduction in NO levels across the tested formulations compared to the positive control (Figure 8). The SJ at the lower concentrations of 1.25 and 2.5 mg/mL did not significantly affect LPS-stimulated NO production compared with the positive control. However, at the higher concentrations of 5 and 10 mg/mL, SJ demonstrated highly significant NO suppression of 60% and 80%, respectively, and can therefore be considered anti-inflammatory. After SGID, the gastric metabolite SJ-G showed anti-inflammatory activity only at 5 mg/mL (60% reduction in NO). SJ-GI did not show any significant effect at either tested concentration (1.25 or 2.5 mg/mL). For the whole metabolites, WJ-G and WJ-GI, a significant anti-inflammatory effect was observed at 2.5 mg/mL, with LPS-stimulated NO production reduced by 38% and 57%, respectively. The anti-inflammatory activity further increased at higher concentrations. At all tested concentrations, WJ-GI demonstrated greater anti-inflammatory potency than WJ-G. This effect peaked at a concentration of 5 mg/mL, where WJ-GI reduced NO production by 80%. In contrast, WJ-G required a higher concentration of 10 mg/mL to achieve the same level of NO reduction.

#### 3.5.4. Effect on Caco-2 Integrity and Permeability

**Figure 9:**
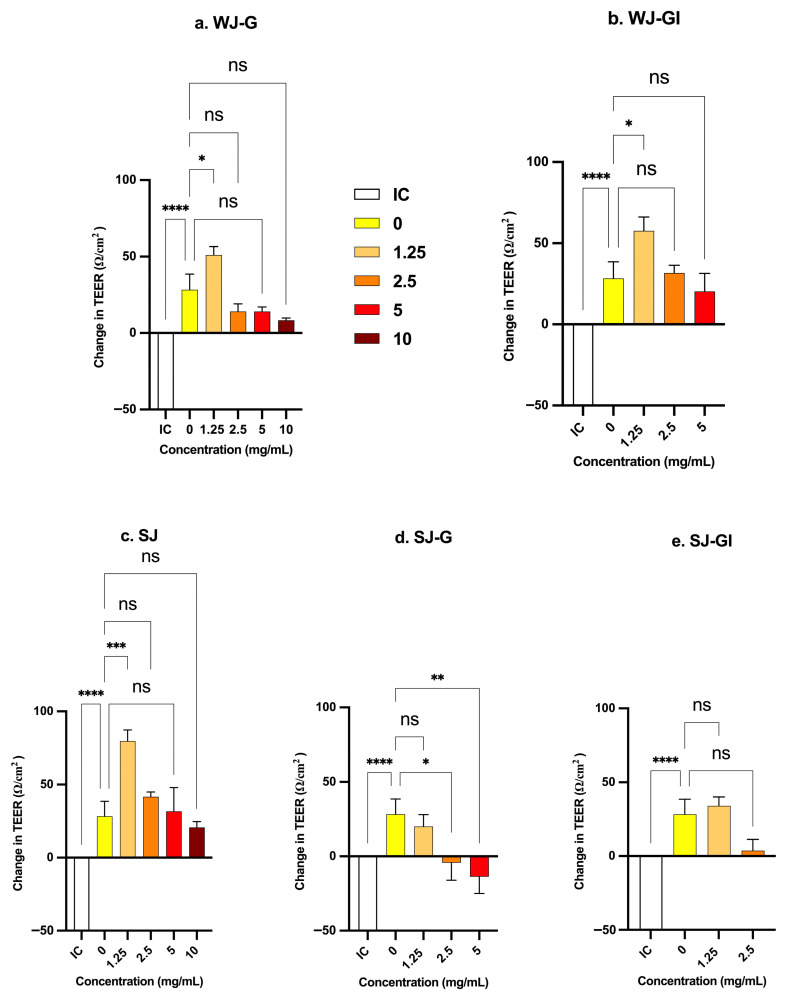
Effect of JEM formulations on the TEER of the Caco-2 cell monolayer. The values are compared to the negative control (0 mg/mL, untreated Caco-2 cells) and the positive control (IC, Caco-2 cells treated with 1 µM ionomycin). Changes in TEER (Ω·cm^2^) reflect positive or negative alterations in epithelial barrier integrity. Significant differences between treatments and control are marked (*, *p* < 0.05, **, *p* < 0.01; ***, *p* < 0.001; ****, *p* < 0.0001). ns: not significant (*p* > 0.05). Error bars denote the standard deviation from the mean of three independent experiments, with each experiment performed in triplicate.

Effect on TEER: The impact of the JEM formulations on the TEER of Caco-2 cell monolayers was evaluated after 24 h of treatment at concentrations of 1.25, 2.5, 5, and 10 mg/mL. In the negative control wells (treatment concentration: 0 mg/mL), TEER increased by 28 ± 10 Ω/cm^2^ over 24 h as the cells differentiated under culture conditions. The newly developed JEM formulations showed concentration-dependent effects, with lower concentrations generally enhancing barrier integrity (increased TEER). At lower SJ concentrations, TEER increased by 79.6 Ω/cm^2^ at 1.25 mg/mL, 41.6 Ω/cm^2^ at 2.5 mg/mL, and 31.6 Ω/cm^2^ at 5 mg/mL. At the highest SJ concentration (10 mg/mL), TEER reached 20.6 Ω/cm^2^ compared to 28 Ω/cm^2^ of the negative control; however, this difference was not statistically significant. However, post-gastric digestion (SJ-G) reduced TEER, peaking at a decrease of 13 Ω/cm^2^ at 5 mg/mL. Whole metabolites (WJ-G and WG-GI) enhanced TEER at low concentrations (51 Ω/cm^2^ and 57.6 Ω/cm^2^, respectively, at 1.25 mg/mL) and showed non-significant differences at higher concentrations compared to controls. Across all formulations, the extent of TEER enhancement decreased with increasing concentration, suggesting a concentration-dependent effect on Caco-2 monolayer integrity (Figure 9).

Effect on paracellular permeability: The effect of JEM formulations on the transport of FITC-Dextran across the Caco-2 monolayer was evaluated by measuring fluorescence in the basolateral compartment after 24 h of exposure to the formulations at various concentrations (1.25, 2.5, 5 and 10 mg/mL). The data are presented as changes in fluorescence from 0 to 24 h post-treatment relative to the negative control (0 mg/mL). The fluorescence changes across different concentrations did not differ significantly (ns), although a non-significant upward trend was observed at higher concentrations (Supplementary Figure S3).

## 4. Discussion

This study reports the first use of high-efficiency jet-O-mizer ultra-fine milling to reduce eggshell membrane (ESM) particle size from an average of 185 μm (from sieve shaker data) to 5.7 μm, followed by KOH-mediated hydrolysis to liberate bioactive compounds, enabling their evaluation for gut health applications. Quality control assessment demonstrated that JEM had very low bacterial contamination (aerobic colony count, total coliforms, *E. coli*) and was free of pathogenic *Salmonella* spp. (Supplementary Table S2). Furthermore, JEM consists primarily of protein (82.9%), suggesting that it is suitable as a safe, high-protein material for dietary applications. Moreover, our previous study demonstrated that reducing ESM particle size enhances its anti-inflammatory and antimicrobial activity [14].

Increasing ESM solubility is crucial for improving its bioavailability and potential as a dietary supplement. Previous methods, including acid or alkali treatments and fermentation with lactic acid bacteria, have shown promise in enhancing ESM solubility and bioactive properties [2,30]. For example, Pasarin et al. (2023) achieved 14.23% hydrolysis using NaOH and Alcalase protease [31], while ultrasonic pre-treatment has been shown to enhance enzymatic hydrolysis efficiency [32].

The primary objective of the current study was to hydrolyze ESM to enhance protein solubility and bioavailability, which we achieved by combining mechanical size reduction (Jet-O-mizing) with chemical hydrolysis using KOH. Based on preliminary data, an optimized protocol was developed to hydrolyze JEM, yielding the highest levels of soluble peptides and a subsequent increase in antioxidant activity (protocol subject to an invention disclosure). The process successfully produced soluble proteins and peptide fractions, yielding approximately 50% protein liberation at lower molecular weights, as verified by SDS-PAGE. The subsequent SGID of the two forms of the JEM hydrolysates (the whole WJ and the soluble/supernatant SJ) provided insights into their biological stability and the extent of further peptide release. WJ formulations released additional soluble peptides during both the gastric and intestinal phases, while SJ formulations showed less change, likely due to pre-digestion solubilization. The desalting step ensured the removal of residual salt formed upon neutralization of the hydrolysate after alkaline hydrolysis, which stabilized the hydrolysate and metabolites and made them suitable for downstream bioactivity assays, establishing a reproducible protocol that can be scaled up for industrial applications.

Our study evaluated the antibacterial activity of SJ against the gut-associated pathogen *E. coli*, building on our previous findings of PEM antimicrobial activities [14]. SJ exhibited a bacteriostatic effect, inhibiting *E. coli* growth by 50% at 10 mg/mL. The antibacterial potential of the developed JEM formulations may be attributed to the liberated peptides and to enhanced bioavailability resulting from alkaline hydrolysis. In the current study, a relatively small number of proteins (39) were identified in JEM-derived formulations (SJ and WJ-G), in contrast to earlier proteomic studies, which reported substantially higher total protein numbers, ranging from 110 to ~500 [5,14]. However, mild and brief sequential washing conditions liberate 69 proteins (water), 17 proteins (NaCl), or 41 proteins (NaOH, 1.25 N, 50 °C) [5]. In the current work, extensive alkaline hydrolysis shifted the soluble protein population into the low-molecular-weight peptide range, with formation of degraded species that are less amenable to confident identification due to suboptimal peptide length, reduced sequence coverage, and limited database matching efficiency, ultimately resulting in fewer identifiable proteins by LC–MS/MS. Comprehensive proteomic interrogation of SJ and WJ-G in this study identified various proteins, including ovocalyxin 36 (OCX36), ovocleidin 17 (OC-17), BPI-fold containing family B member 2 (BPIFB2, TENP), ovalbumin, and the histone variants H2AC39 and H2BC32. Antimicrobial proteins are significant constituents of the ESM matrix, including OC-17, OCX36, defensins, lysozyme, ovoinhibitor, ovostatin, ovalbumin-related protein-X (OVAX), and ovotransferrin [33]. OCX-36 exhibits a high affinity for bacterial pyrogens, including LPS and lipoteichoic acid (LTA) [5]. ESM is naturally enriched with antimicrobial proteins and peptides, including lysozyme, histones, avian β-defensins (AvBDs), and ovalbumin [5,7]. Additionally, glycoproteins such as ovomucin and mucin have antibacterial and antiviral properties [34,35]. Similarly, ESM hydrolysates demonstrated antimicrobial activity against a broad spectrum of pathogens, including *S. aureus*, *B. subtilis*, *K. pneumoniae*, *S. marcescens*, and *E. coli* [36]. Moreover, peptide derivatives of ESM histones retain antimicrobial activity [5,14]. These findings highlight the potential of JEM formulations in gut health applications, particularly for managing dysbiosis-associated disorders.

Oxidative stress is a key factor in the onset of various GI disorders as it can harm cellular structures, promote inflammation, and contribute to diseases like IBD and colorectal cancer [37]. Safe antioxidants from natural, accessible sources are alternatives that have attracted widespread interest among researchers. Protein hydrolysates exhibit better antioxidant activity than their intact forms [38]. Ultrafiltered ESM enzymatic hydrolysate fractions demonstrated a range of antioxidant activity, including iron (Fe^3+^) reducing, 2,2-diphenyl-1-picrylhydrazyl (DPPH), hydroxyl radical scavenging, and Fe^2+^ chelating activity, which were further verified by a cell-based study that ESM hydrolysate reduced proinflammatory cytokine IL-8 secretion in oxidative stress-induced human intestinal epithelial Caco-2 cells [39]. Similarly, ESM enzymatic hydrolysates and corresponding identified synthetic peptides showed a strong ability to quench ABTS, inhibit TBARS and high total antioxidant activity in a study by Zhao and coworkers [40].

In this study, we evaluated the antioxidant power of the soluble proteins liberated upon JEM alkaline hydrolysis, followed by SGID, using the Trolox equivalent antioxidant capacity assay. SJ showed antioxidant activity up to 15 times greater than NJEM, peaking at 667 µM Trolox equivalents at 10 mg/mL. This antioxidant capacity was retained after digestion, demonstrating the potential to mitigate oxidative stress in biological systems. Interestingly, functional annotation clustering of proteins identified in SJ, performed using DAVID bioinformatics resources, revealed enrichment for the peroxidase activity GO term (GO:0,004,601, six proteins), along with all potential protein–protein interactions among them, as determined by STRING analysis (Figure 3). This is consistent with a study indicating that hemoglobin may play a role in protecting against oxidative damage [41]. Moreover, Hb exhibits pseudoperoxidase activity, enabling it to decompose hydrogen peroxide (H_2_O_2_) and potentially mitigate oxidative stress [42]. In addition, hemoglobin can mitigate H_2_O_2_ oxidative stress by acting as an antioxidative peroxidase [43]. Finally, ESM comprises proteins such as CREMP, ovalbumin, ovotransferrin, and cystatin that contain amino acids which possess antioxidant activities, such as cysteine, histidine, tryptophan, and β-hydroxyl tryptophan [18,44,45,46]. Therefore, we propose that the hydrolysis of cysteine-rich CREMPs and other ESM proteins contributed to the enhanced antioxidant activity of JEM formulations.

Inflammation plays a pivotal role in the development of numerous gut disorders [47]. In a murine dextran sodium sulfate-induced colitis model, dietary ESM improved inflammatory mediator gene expression and regulated Th17 cell expansion, thereby reducing mucosal inflammation [48]. ESM improves microbial alpha diversity and reduces inflammation-associated microbiota, while increasing total organic acid levels, particularly SCFAs such as butyrate (2.3-fold), which are known to inhibit Th1 and Th17 production [20]. A form of particalized ESM (PEM < 53 µm) exhibited a 56 ± 7% reduction in LPS-stimulated NO levels after 24 h [49], compared to the 80 ± 0.6% reduction achieved with KOH-hydrolyzed JEM in the current study.

This study assessed the anti-inflammatory effects of JEM formulations using LPS-induced RAW 264.7 macrophages. The focus was on modulating NO production, a key marker of inflammation, via TLR4-mediated signaling pathways [50]. Optimal LPS concentration (1, 5, 10, and 20 µg/mL) was evaluated in RAW 264.7 macrophages, where 1 µg/mL was observed to induce NO production similar to 20 µg/mL, but without affecting viability. According to ISO 10993-5, cell viability above 80% is defined as non-cytotoxic [51]. Thus, JEM formulations (1.25–10 mg/mL) were non-toxic, maintaining >80% cell viability. JEM showed dose-dependent anti-inflammatory activity, achieving up to 80% suppression of NO production at 10 mg/mL. Post-gastrointestinal digestion products retained potent anti-inflammatory effects, highlighting the stability and bioactivity of hydrolyzed peptides under physiological conditions. Metabolites of the whole JEM hydrolysate (WJ: WJ-G and WJ-GI) showed the most significant anti-inflammatory effects, possibly due to the presence of diverse bioactive peptides generated during the digestion of WJ. Proteomic analysis identified multiple proteins with demonstrated anti-inflammatory activities, including TENP, OCX36 in both SJ and WJ-G, in addition to tyrosine 3-monooxygenase/tryptophan 5-monooxygenase activation protein epsilon (YWHAE) in SJ and Y-box binding protein 1 (YBX1) in WJ-G (Table 2). In addition, functional annotation clustering of proteins identified in SJ and WJ-G revealed enrichment for the response to corticosterone GO term (GO:0,051,412, 2 proteins; TENP and OVAL), along with their potential protein–protein interactions as determined by STRING analysis (Figure 3). In addition to its antimicrobial activity, OCX36 binds LPS and lipoteichoic acid (LTA) to neutralize endotoxins, and both OCX36 and digested OCX36 (dOCX36) modulate immune responses. In mice, dOCX36 reduced LPS-induced inflammation more effectively than OCX36 and suppressed local pro-inflammatory mediators [5]. Accumulating evidence indicates that the OCX36 and TENP genes are evolutionarily conserved and functionally aligned, contributing to innate immune surveillance and modulating host inflammatory responses [33]. Additionally, YWHAE, as a key TNFR2 complex component, modulates macrophage polarization via PI3K/Akt/mTOR, restricting NF-κB activation and promoting anti-inflammatory responses, protecting against inflammation and autoimmune pathology [52]. Furthermore, YBX1 interferes with TNFα receptor binding in a dose-dependent manner and modulates TNFα receptor signaling and downstream responses, demonstrating immunoregulatory activity that indirectly attenuates TNFα-driven inflammatory responses [53]. The findings from this model provide valuable insights into the potential of JEM formulations as anti-inflammatory agents against gut-related disorders. Anti-inflammatory and antimicrobial proteins collectively shape intestinal homeostasis by dampening mucosal immune activation, reinforcing epithelial barrier integrity, and modulating host–microbe interaction that determines microbial composition and function; these coordinated actions influence community structure, metabolic output, and resilience of the gut ecosystem [54].

The integrity of the intestinal barrier is essential for maintaining its function, and its disruptions have been linked to various diseases [55], including IBD [56], irritable bowel syndrome (IBS) [57] and metabolic syndrome [58]. Previous *in vitro* and *in vivo* studies showed the impact of proteins and derived bioactive peptides on regulating intestinal physical, chemical, biological and immunological barrier function [59], such as fish protein hydrolysate [60], soybean β-conglycinin hydrolysate [61], and wheat gluten exploring A5 and C5 [62]. Alaska pollock skin-derived collagen and its tryptic hydrolytic fractions significantly mitigated TNFα-induced barrier dysfunction in a Caco-2 cell monolayer by alleviating disruption of tight junction proteins ZO-1 and occludin, while also inhibiting MLC phosphorylation and MLCK expression, and suppressing the activation of NFκB and Elk-1 [63]. Casein hydrolysate and derived peptides improved intestinal barrier function in diabetes-prone (DP) BioBreeding (BB) rats, as evidenced by a decrease in the lactulose: mannitol ratio, reduced serum zonulin levels, and increased ileal TEER [64].

Here, we used differentiated Caco-2 cells to study the effect of our developed JEM formulations on a model of intestinal barrier function, by evaluating TEER and paracellular tracer (FITC-Dextran) transport. Our results revealed that a low concentration (1.25 mg/mL) of the soluble portion of JEM hydrolysate (SJ) and the metabolites of the whole hydrolysate (WJ-G and WJ-GI) significantly increased TEER, indicating enhanced intestinal barrier integrity, whereas higher concentrations did not have a significant effect on TEER. Fluorescence analysis of the basolateral compartment showed no significant differences in FITC-Dextran transport across Caco-2 monolayers treated with JEM formulations after 24 h. These *in vitro* findings indicate that JEM formulations do not disrupt paracellular intestinal permeability or integrity, even at the highest concentration of 10 mg/mL, at which the greatest anti-inflammatory effect was observed.

The results of this study have broader implications for the utilization of ESM as a sustainable, functional ingredient in health-focused applications. With millions of tons of ESM generated annually as an industrial by-product, its transformation into value-added products aligns with global efforts to reduce waste and promote circular economies. The compositional richness of ESM, including its collagen content and bioactive peptides, positions it as a versatile material for nutraceuticals, pharmaceuticals, and functional foods. The demonstrated antioxidant, antimicrobial, and anti-inflammatory activities of JEM formulations highlight their potential as dietary supplements to boost gut health.

## 5. Conclusions

The current study demonstrated an innovative, scalable strategy to valorize eggshell membrane (ESM) into bioactive-rich formulations with potential for gut health applications. High-efficiency jet-O-mizer processing reduced average ESM particle size to 5.7 µm, enabling enhanced accessibility for subsequent KOH-mediated hydrolysis, which achieved ~50% liberation of low-molecular-weight peptides. The resulting soluble fraction (SJ) exhibited markedly enhanced functional properties, including up to 15-fold higher antioxidant capacity compared to unhydrolyzed ESM, with activity retained following simulated gastrointestinal digestion. JEM-derived formulations exhibited significant bioactivities. SJ exerted bacteriostatic effects against E. coli (50% inhibition at 10 mg/mL), while both SJ and digestion-derived metabolites (WJ-G, WJ-GI) displayed potent anti-inflammatory activity, suppressing LPS-induced nitric oxide production in RAW 264.7 macrophages by up to 80% without cytotoxicity. Proteomic analysis identified key antimicrobial and immunomodulatory proteins (e.g., OCX36, TENP, YWHAE, and YBX1) that could support immune modulation and redox regulation. Functional annotation further indicated enrichment in peroxidase-related activity, consistent with observed antioxidant effects. Importantly, JEM formulations enhanced intestinal barrier integrity in differentiated Caco-2 monolayers, increasing TEER at low concentrations without compromising paracellular permeability. Collectively, these findings demonstrate that combining ultra-fine milling with alkaline hydrolysis generates stable, bioaccessible peptide fractions with integrated antioxidant, antimicrobial, anti-inflammatory, and barrier-supportive functions. This work extends ESM utilization into gastrointestinal health applications and provides a robust platform for developing sustainable, clinically translatable nutraceuticals and functional ingredients.

## 6. Limitations

This study is limited by its dependence on *in vitro* models, which do not fully capture the complexity of *in vivo* gastrointestinal systems, including host–microbiota interactions and the associated immunomodulation. Antimicrobial activity was assessed using a single representative strain (*E. coli* O157), which may limit generalization. Extensive alkaline hydrolysis, while effective for peptide release, reduced proteomic detectability, making full identification of all bioactive components challenging. Mechanistic insights were inferred from functional and proteomic analyses but have not yet been confirmed with targeted molecular studies. Future *in vivo* validation, microbiome analysis, upscaling, dose optimization, and clinical investigation to confirm translational relevance are necessary. 

## Figures and Tables

**Table 1:** The change in soluble protein content of SJ and WJ upon SGID.

Concentration of Soluble Proteins and Peptides (mg/mL)		
	**WJ**	**SJ**
	22.9 ± 4.4	22.9 ± 4.4
**SGD**		
Initial	11.5	11.5
Final	21.6 ± 0.4	11.2 ± 0.6
**SID**		
Initial	10.5	5.5
Final	14 ± 1.3	5 ± 1.5

Summary of the concentrations of liberated soluble proteins and peptides measured by the BCA assay of SJ and WJ throughout SGID.

**Table 2:** Comparison of SJ and WJ-G proteomic inventories.

No.	Protein Name	Gene ID	Official Gene Symbol	No. of Unique Peptides	
				SJ	WJ-G
1	3-hydroxymethyl-3-methylglutaryl-CoA lyase	396316	HMGCL	-	1
2	Actin gamma 1 like	415296	ACTG1L	1	-
3	Apovitellenin 1	396476	APOV1	1	-
4	BPI fold containing family B member 2 (TENP)	395882	BPIFB2	2	2
5	Calmodulin 1 (phosphorylase kinase, delta)	396523	CALM1	-	1
6	Collagen type X alpha 1	100858979	COL10A1	2	1
7	CREMP	107049904	LOC107049904	11	11
8	Envoplakin	427805	EVPL	-	1
9	Exostosin glycosyltransferase 2	425859	EXT2	-	1
10	Fatty acid binding protein 3	419557	FABP3	1	1
11	Fatty acid binding protein 7	396246	FABP7	1	1
12	Hemoglobin alpha, subunit D	416651	HBAD	1	-
13	Hemoglobin beta, subunit rho	419079	HBBR	2	-
14	Hemoglobin subunit alpha 1	416652	HBA1	1	1
15	Hemoglobin subunit epsilon	107049060	HBE	1	-
16	Hemoglobin subunit epsilon 1	396485	HBBA	2	3
17	Hemoglobin subunit zeta	416650	HBZ	1	-
18	Histone H2A-IX (H2A clustered histone 39)	417947	H2AC39	1	-
19	Histone H2B-II (H2B clustered histone 32)	100858607	H2BC32	1	1
20	Keratin 5	407779	KRT5	1	2
21	Keratin 7	395772	KRT7	1	-
22	Keratin 24	395861	KRT24	1	-
23	Matrix extracellular phosphoglycoprotein	395256	MEPE	1	2
24	Mitochondrial ribosomal protein L40	100858795	MRPL40	1	-
25	Motile sperm domain containing 2	418627	MOSPD2	1	-
26	Myosin light chain kinase	396445	MYLK	-	1
27	Myristoylated alanine rich protein kinase C substrate	396473	MARCKS	1	1
28	Nucleosome assembly protein 1 like 1	417864	NAP1L1	-	1
29	Ovalbumin (SERPINB14)	396058	OVAL	1	4
30	Ovocalyxin-36 (BPI fold containing family B member 3)	419289	BPIFB3	2	2
31	Ovocleidin 17 (OC-17)	121109246	OC17	1	2
32	Phosphodiesterase 6D	424932	PDE6D	-	1
33	Proteasome subunit alpha 1	395874	PSMA1	-	1
34	Pyruvate kinase, liver and RBC	396456	PKLR	1	-
35	Rho GTPase activating protein 17	416564	ARHGAP17	-	1
36	Serine and arginine rich splicing factor 2	396195	SRSF2	1	-
37	Small nuclear ribonucleoprotein polypeptide F	417916	SNRPF	1	-
38	Tyrosine 3-monooxygenase/tryptophan 5-monooxygenase activation protein epsilon	417554	YWHAE	1	-
39	Y-box binding protein 1	386575	YBX1	-	2

**Table 3:** Functional annotation analysis of the protein constituents identified in SJ and WJ-G samples.

Cluster No.	Go Terms	No. of Proteins	Official Gene Symbol
1	GO:0,004,601 Peroxidase activity	6	*HBAD*, *HBBR*, *HBA1*, *HBE*, *HBBA*, *HBZ*
2	GO:0,008,289 Lipid binding	4	*BPIFB2*, *BPIFB3*, *FABP3*, *FABP7*
3	GO:0,046,982 Protein heterodimerization activity	4	*H2AC39*, *H2BC32*, *EXT2*, *YWHAE*
4	GO:0,045,109 Intermediate filament organization	3	*KRT5*, *KRT7*, *KRT24*
	GO:0,005,198 Structural molecule activity	3	*EVPL*, *HMGCL*, *KRT24*
5	GO:0,031,215 Shell calcification	2	*MEPE*, *OC17*
6	GO:1,905,913 Negative regulation of calcium ion export across plasma membrane	2	*CALM1*, *YWHAE*
7	GO:0,051,412 Response to corticosterone	2	*BPIFB2*, *OVAL*
8	GO:2,000,767 Positive regulation of cytoplasmic translation	2	*PKLR*, *YBX1*

**Table 4:** Bioactivities of JEM formulations.

JEM Formulation	Conc.	1. TEAC	2. Anti-Inflammatory Activity	3. Change in TERR	4. FITC Transport	5. Antibacterial Activity
SJ	1.25	236 *** ± 50	126 ± 17	80 *** ± 8	2.3 ± 0.6	22% ***
	2.5	361 **** ± 3	111 ± 5	42 ± 3	2.7 ± 0.6	27% ****
	5	363 **** ± 51	42 ** ± 13	32 ± 16	2.7 ± 0.6	33% ****
	10	667 **** ± 25	20 **** ± 1	21 ± 4	3 ± 1	50% ****
SJ-G	1.25	160 **** ± 4	96 ± 10	20 ± 8	2.3 ± 0.6	
	2.5	272 **** ± 8	78 ± 7	−4 * ± 12	2.3 ± 0.6	
	5	377 **** ± 10	39 *** ± 8	−13 ** ± 11	4.7 ± 1.2	
SJ-GI	1.25	186 *** ± 34	122 ± 27	34 ± 6	4.7 ± 0.6	
	2.5	274 **** ± 21	107 ± 10	4 ± 8	5.3 ± 0.6	
WJ-G	1.25	144 **** ± 13	89 ± 2	51 * ± 6	1.7 ± 0.6	
	2.5	237 **** ± 17	62 ** ± 10	14 ± 5	2.3 ± 0.6	
	5	381 **** ± 6	35 *** ± 5	14 ± 3	2.7 ± 0.6	
	10	698 **** ± 15	19 **** ± 2	8 ± 11	3.3 ± 0.6	
WJ-GI	1.25	220 **** ± 41	80 ± 2	58 * ± 9	1.7 ± 0.6	
	2.5	325 **** ± 18	43 *** ± 8	32 ± 5	2.3 ± 0.6	
	5	370 **** ± 14	19 **** ± 2	20 ± 11	2.7 ± 0.6	

1. Antioxidant activity (Trolox equivalent antioxidant capacity, TEAC, uM). 2. Anti-inflammatory activity (% inhibition of NO production in LPS-induced RAW 264.7 RAW macrophages). 3. Effect on intestinal barrier integrity (change in TEER of Caco-2 cell monolayer after 24 h of treatment; positive values indicate an increase, and negative values indicate a decrease in TEER). 4. FITC transport (Paracellular Permeability Assessment, change in fluorescence (A.U.) detected in the basolateral compartment of a Caco-2 monolayer model after 24 h of treatment). 5. Antibacterial activity (percentage inhibition of growth of *E. coli*, at 24 h). Significant differences between treatments and control are marked (*, *p* < 0.05; **, *p* < 0.01; ***, *p* < 0.001; ****, *p* < 0.0001).

## Data Availability

The original contributions presented in this study are included in the article/Supplementary Material. Further inquiries can be directed to the corresponding author.

## Supplementary Materials

The following supporting information can be downloaded at: https://www.mdpi.com/article/10.3390/molecules31071217/s1, Supplementary Table S1. The chemical composition of eggshell membrane (ESM). Supplementary Table S2. The microbiological profile of JEM. Supplementary Table S3. The chemical composition of jet-O-mized eggshell membrane (JEM). Supplementary Figure S1. Particle Size Distribution of ESM powder after jet-O-mizing (JEM), in 2-propanol, analyzed by Beckman Coulter LS Particle Size Analyzer. Supplementary Figure S2. A: SDS-PAGE analysis showing the release of soluble proteins from JEM during alkaline hydrolysis. The gel displays samples collected at 0, 24, and 48 hours, each indicating the progression of protein solubilization. B: SDS-PAGE gel analysis showing the release of a soluble protein smear upon SGID of SJ and WJ formulations. Each lane represents a different time point and amount of sample loaded (measured by BCA assay after desalting). Protein marker (kDa) in the center lane provides MW standards from 3.5 kDa to 260 kDa. Supplementary Figure S3. The effect of JEM formulations on the transport of FITC-CM-Dextran (FD-4) across the Caco-2 cell monolayer is expressed as a change in fluorescence (A.U) in the basolateral compartment after 24 hours of treatment. The negative control (Concentration 0 mg/mL) is untreated Caco-2 cells, and the positive control is Caco-2 treated with 1 µM ionomycin (IC). Higher fluorescence in the basolateral compartment indicates enhanced transport of Dextran-FITC. Significant differences between treatments and control are marked (** *p* < 0.01, *** *p* < 0.001, **** *p* < 0.0001). Error bars denote the standard deviation from the mean of three independent experiments.

## Abbreviations

The following abbreviations are used in this manuscript:

ABTS	2′,2′-Azino-bis(3-ethylbenzothiazoline-6-sulfonic acid)
ANOVA	Analysis of variance
BCA	Bicinchoninic acid
CCM	Complete culture media
CFU	Colony forming unit
CREMP	Cysteine-rich eggshell membrane protein
CRP	C-reactive protein
DAVID	Database for annotation, visualization and integrated discovery
DMEM	Dulbecco’s Modified Eagle Medium
DPPH	2,2-diphenyl-1-picrylhydrazyl
EDTA	Ethylenediaminetetraacetic acid
ES	Eggshell
ESM	Eggshell membrane
FD-4	Fluorescein isothiocyanate–carboxymethyl–dextran
FDR	False discovery rate
FITC	Fluorescein isothiocyanate
GO	Gene Ontology
H_2_O_2_	Hydrogen peroxide
HA	Hyaluronic acid
HJEM	Hydrolyzed jet-O-mized eggshell membrane
HPEM	Hydrolyzed particalized eggshell membrane
IBD	Inflammatory bowel disease
IBS	Irritable bowel syndrome
IC	Ionomycin
IL-10	Interleukin 10
IL-1β	Interleukin 1β
JEM	Jet-O-mized eggshell membrane
KCl	Potassium chloride
KOH	Potassium hydroxide
LC/MS/MS	Liquid chromatography–tandem mass spectrometry
LPS	Lipopolysaccharides
NaCl	Sodium chloride
NaOH	Sodium hydroxide
NC	Negative control
NJEM	Non-hydrolyzed jet-O-mized eggshell membrane
NO	Nitric oxide
OD	Optical density
PEM	Particalized eggshell membrane
RPM	Round per minute
SCFA	Short-chain fatty acids
SDS-PAGE	Sodium dodecyl sulfate-polyacrylamide gel electrophoresis
SGD	Simulated gastric digestion
SGID	Simulated gastrointestinal digestion
SID	Simulated intestinal digestion
SIF	Simulated intestinal fluid
SJ	Soluble jet-O-mized eggshell membrane
SJ-G	Soluble jet-O-mized eggshell membrane-gastric digestion
SJ-GI	Soluble jet-O-mized eggshell membrane-gastrointestinal digestion
TBARS	Thiobarbituric acid reactive substances
TEAC	Trolox equivalent antioxidant capacity
TEER	Transepithelial electrical resistance
TNFα	Tumor necrosis factor α
WJ	Whole hydrolysate of jet-O-mized eggshell membrane
WJ-G	Whole hydrolysate of jet-O-mized eggshell membrane-gastric digestion
WJ-GI	Whole hydrolysate of jet-O-mized eggshell membrane-gastrointestinal digestion
