# Supplementary material for: Scalable Jet-O-Mized Eggshell Membrane Processing for Bioactives Promoting Gut Health
Source: Molecules. 2026 Apr 7;31(7):1217. doi: 10.3390/molecules31071217 (PMC13074765; doi:10.3390/molecules31071217)
Supplement: Supplementary file 1 [file molecules-31-01217-s001.zip › molecules-4167731-supplementary.pdf]

molecules

MDPI

Supplementary Materials

Scalable Jet-O-Mized Eggshell Membrane Processing for Bioactives Promoting Gut Health

Manar Younes [1], Tamer A. E. Ahmed [1], Riadh Hammami [2] and Maxwell T. Hincke [1,3,*]

[1] Department of Cellular and Molecular Medicine, Faculty of Medicine, University of Ottawa, Ottawa, ON K1H 8M5, Canada; myoun141@uottawa.ca (M.Y.); tahmed@uottawa.ca (T.A.E.A.)
[2] School of Nutrition Sciences, Faculty of Health Sciences, University of Ottawa, Ottawa, ON K1H 8M5, Canada; riadh.hammami@uottawa.ca
[3] Department of Innovation in Medical Education, Faculty of Medicine, University of Ottawa, Ottawa, ON K1H 8M5, Canada
* Correspondence: mhincke@uottawa.ca; Tel.: +1-613-562-5800 (ext. 8193)

Supplementary Table S1. The chemical composition of eggshell membrane (ESM).

No.	Chemical composition (%)	Constituents (Percentage)
1.	Protein (90%)	LOXL2: Lysyl oxidase-like 2 (25.2%)<br>CREMPs: Cysteine-rich eggshell membrane proteins (24.3%)<br>LYZ: Lysozyme (10.8%)<br>Collagen X (9.0%)<br>Others (~500 proteins) (20.7%)
2.	Carbohydrate (2%)	HA: Hyaluronic acid<br>KS: Keratan sulfate<br>CS: Chondroitin sulfate<br>HS: Heparan sulfate
3.	Lipids (3%)	
4.	Water/minerals (5%)	

Supplementary Table S2. The microbiological profile of JEM.

ESM POWDER AFTER JET-O-MIZER (5 sub-samples@ 150 grams each)		
Aerobic colony count	<5	CFU/g
Total coliforms	<10	CFU/g
Escherichia coli	<10	CFU/g
Salmonella spp - Detection - MFLP-29	Negative	-

Microbiological assessment of JEM powder (performed by Eurofins Environex, Quebec, Canada).

Supplementary Table S3. The chemical composition of jet-O-mized eggshell membrane (JEM).

5527579 /	ESM-POWDER AFTER JET-O-MIZER (5 sub-samples @ 150 grams each)		
	Total fat	0.78	g/100g
	Saturated fat	0.26	g/100g
	Monounsaturated fat	0.36	g/100g
	Polyunsaturated fat	0.11	g/100g
	Trans fat	0.01	g/100g
	Omega 3	<0.01	g/100g
	Omega 6	0.10	g/100g
	Nitrogen	13.26	%
	Protein	82.90	%
	Ash	15.8	%
	Carbohydrate	<0.00	%
	Energy (Cal/100g)	339	Cal/100g
	Energy (KJ/100g)	1417	KJ/100g
	Energy from fat (KJ/100g)	29	KJ/100g
*	Glucosamin	annexe	%
*	Chondroitine sulfate	annexe	%
*	Collagen	Annexe	-
*	Hyaluronique acid	annexe	-
	Humidity	4.61	%
Remarks:	Our Scope of Accreditation does not cover this type of sample.		

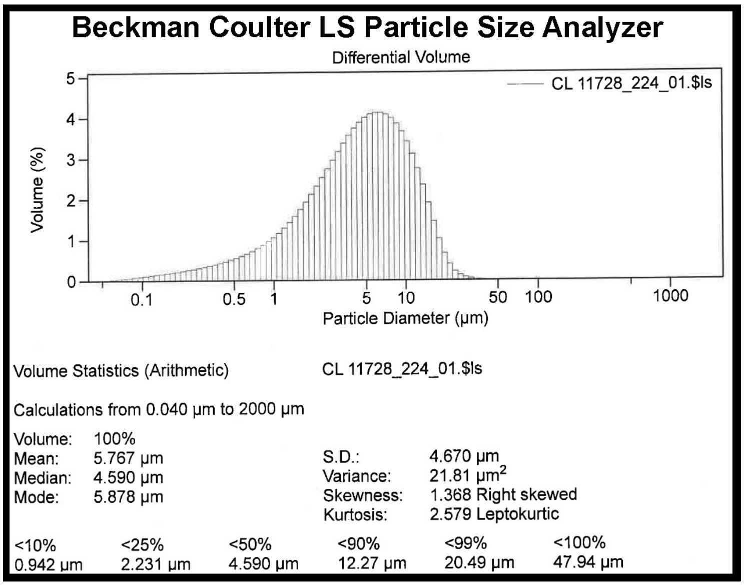

Supplementary Figure S1. Particle Size Distribution of ESM powder after jet-O-mizing (JEM), in 2-propanol, analyzed by Beckman Coulter LS Particle Size Analyzer.

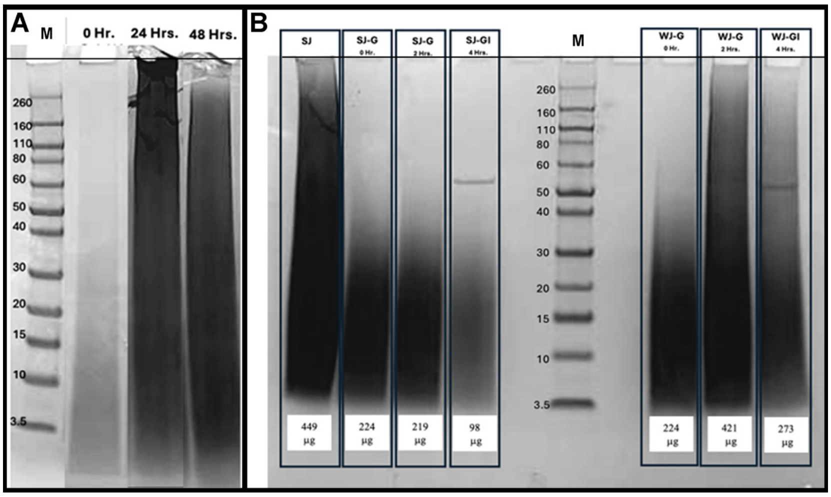

Supplementary Figure S2. **A**: SDS-PAGE analysis showing the release of soluble proteins from JEM during alkaline hydrolysis. The gel displays samples collected at 0, 24, and 48 hours, each indicating the progression of protein solubilization. B: SDS-PAGE gel analysis showing the release of a soluble protein smear upon SGID of SJ and WJ formulations. Each lane represents a different time point and amount of sample loaded (measured by BCA assay after desalting). Protein marker (kDa) in the center lane provides MW standards from 3.5 kDa to 260 kDa.

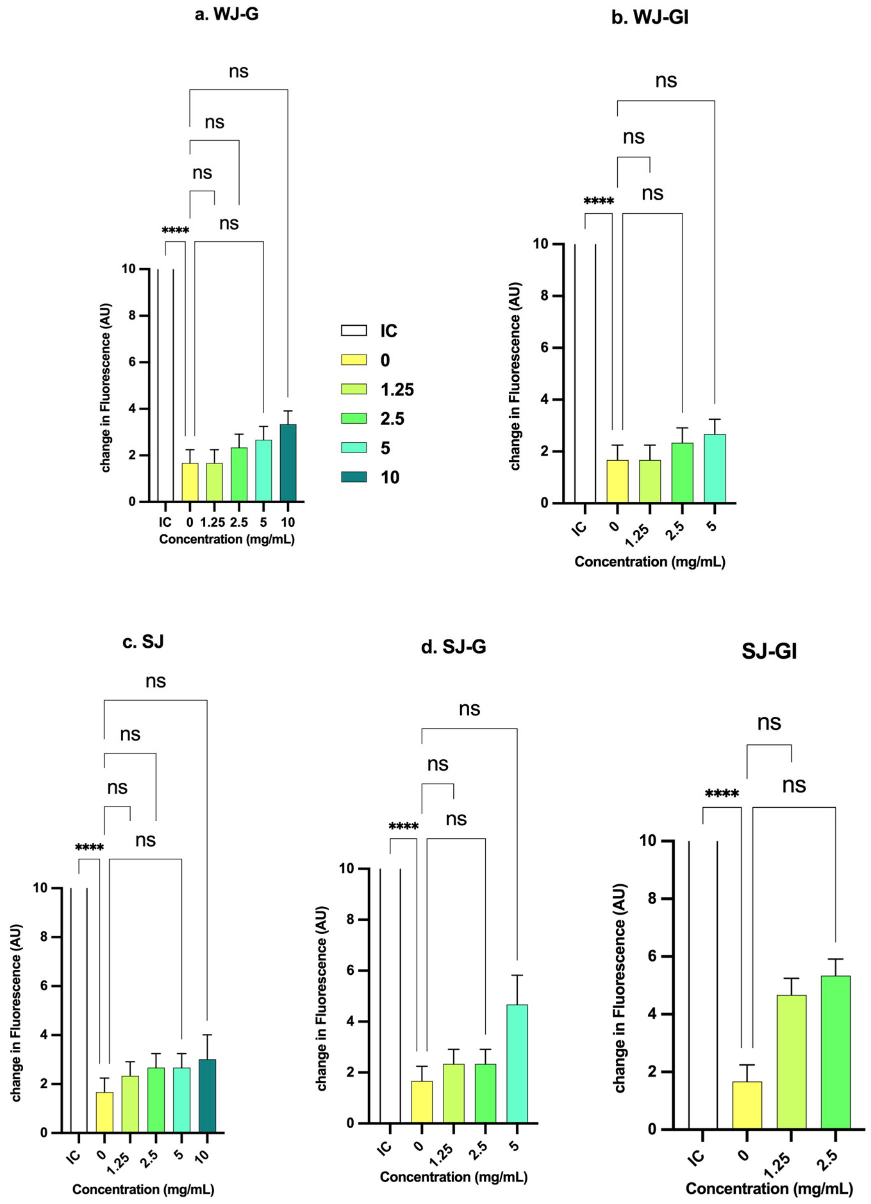

Supplementary Figure S3. The effect of JEM formulations on the transport of FITC-CM-Dextran (FD-4) across the Caco-2 cell monolayer is expressed as a change in fluorescence (A.U) in the basolateral compartment after 24 hours of treatment. The negative control (Concentration 0 mg/mL) is untreated Caco-2 cells, and the positive control is Caco-2 treated with 1 µM ionomycin (IC). Higher fluorescence in the basolateral compartment indicates enhanced transport of Dextran-FITC. Significant differences between treatments and control are marked (** $p < 0.01$, *** $p < 0.001$, **** $p < 0.0001$). Error bars denote the standard deviation from the mean of three independent experiments.
